# Correlation between tobacco smoking and dental caries: A systematic review and meta-analysis

**DOI:** 10.18332/tid/106117

**Published:** 2019-04-19

**Authors:** Xue Jiang, Xiaoge Jiang, Yan Wang, Ruijie Huang

**Affiliations:** 1Department of Pediatric Dentistry, State Key Laboratory of Oral Diseases, National Clinical Research Center for Oral Diseases, West China Hospital of Stomatology, Sichuan University, Chengdu, China

**Keywords:** tobacco smoking, dental caries, meta-analysis

## Abstract

**INTRODUCTION:**

Tobacco is responsible for infaust oral conditions and many oral diseases. Dental caries is one of the most prevalent oral diseases. The association between tobacco smoking and dental caries has become an important recent topic of research. A systematic review and meta-analysis was conducted to evaluate the association between tobacco smoking and dental caries.

**METHODS:**

A literature search was conducted in the databases PubMed, EMBASE, Medline and Cochrane, up to December 2018. Original observational articles that estimated relevance between tobacco smoking and dental caries in adults were included. Caries were determined by measurements of decayed, missing or filled teeth (DMFT), or decayed, missing or filled surface (DMFS), or caries-related microflora levels. Trials did not include a non-smoking group, exposure to smokeless tobacco products, or participants under 16 years old. Also, literature reviews, comments, case reports and letters to the editor were not considered. Both methods of systematic review and meta-analysis were adopted. Newcastle-Ottawa Scale (NOS) was used to assess the methodological quality of all the included studies.

**RESULTS:**

Ten out of eleven of the included studies indicated a positive association between tobacco smoking and dental caries. Two meta-analyses were performed: one included five studies using DMFT as an outcome; the other included two studies of DMFS. A random effects model was used. Both were highly heterogeneous (I^2^=93%, chi-squared p<0.00001; I^2^=70%, chi-squared p=0.07, respectively) and statistically significant (mean difference, MD=1.20, 95% confidence interval, CI: 0.40–2.00, z-test p=0.003; MD=1.88, 95% CI: 0.99–2.77, z-test p<0.0001, respectively). The quality scores of all varied from 7 to 9.

**CONCLUSIONS:**

There is a correlation between tobacco smoking and an increased risk of dental caries. However, the overall representativeness of the studies is not good. More prospective and extensive research on this topic is needed to get validation. Even so, it is imperative that people quit tobacco smoking.

## INTRODUCTION

Tobacco is detrimental to human health. The latest Surgeon General’s Report (SGR), ‘The Health Consequences of Smoking–50 Years of Progress’, updated the evidence of the infaust effects of smoking on health^1^. The World Health Organization (WHO) estimates that tobacco is responsible for more than 7 million deaths and hundreds of billions of dollars loss, worldwide each year^2^. More than 60 toxic chemicals in tobacco such as nicotine can invade the body’s multiple systems^3^. They lead to cardiovascular diseases, cancers and other systemic diseases^4^. In addition, tobacco is a harmful product responsible for adverse oral conditions and some oral diseases. Any form of tobacco consumption is responsible for oral diseases like adult periodontal diseases, oral cancer, cleft lip, cleft palate and other congenital defects in children whose mothers smoked tobacco during pregnancy^5^.

Dental caries is a primary focus of dental health prevention as it is one of the most prevalent chronic diseases^6^, which is only secondary to the flu^7^. It remains a major global health problem that not only affects adults and school-age children but also pre-school children^8^. The severity of dental caries and odontogenic infections varies from mild buccal space infection to severe multi-space infection^9^. If not treated on time, dental caries can cause progressive destruction of tooth hard tissue, perforate into pulp, lead to pulpitis and periapical inflammation, and finally lead to teeth loss^10^. Many factors such as food, environment and microorganisms are associated with caries.

An increasing number of studies have focused on the association between tobacco smoking and dental caries. Early in 1998, a cross-sectional epidemiological study^11^ in Sweden concluded that there was an association between smoking and oral health. Later, more trials emerged, but the conclusions have not been consistent, hence further studies are needed. In the last five years, no systematic review on this topic has been conducted.

A systematic review and meta-analysis has been designed to evaluate the association between tobacco smoking and dental caries. The hypothesis is that tobacco smoking is a risk factor for dental caries.

## METHODS

This review was based on the Preferred Reporting Items for Systematic Review and Meta-Analysis (PRISMA) guidelines^12^.

### Eligibility criteria

The inclusion criteria for studies were: 1) original observational articles that estimated relevance between tobacco smoking and dental caries in adults with a non-smoking group for comparison; 2) determining caries by measuring decayed, missing or filled teeth (DMFT), or decayed, missing or filled surface (DMFS), or caries related microflora levels; and 3) studies that were published in English.

The exclusion criteria were: 1) without a non-smoking group; 2) exposure to smokeless tobacco products; 3) participants younger than 16 years old; and 4) studies characterized as literature reviews, comments, case reports, *in vitro* studies, or letters to the editor.

### Search strategy and studies selection

A thorough electronic search was conducted in the databases PubMed, EMBASE, Medline and Cochrane to identify relevant research. Studies published up to December 2018 were included. The search string was: (Smoking OR Tobacco OR Tobacco smoking OR Tobacco products OR Cigarette smoking OR Cigarette OR Cigar) AND (Dental caries OR Dental decay OR Teeth decay OR Caries). No data and language restrictions were applied in searching.

In the beginning, duplicate articles were excluded. Then titles and abstracts of studies were independently assessed by two authors based on the eligibility criteria. Finally, the full text of articles that were initially included was evaluated according to the inclusion and exclusion criteria. Along the process, the two authors reached consensus through discussion, if their opinions were different. Cohen’s kappa was adopted to assess the inter-reviewer reliability.

### Data extraction

The following elements were extracted from each article: the surname of first author, year of publication, study type, search site, sample size, gender contribution, mean age and age range, exposure assessment, caries evaluation, result, and judgment of irrelevant variable. These data were independently extracted by two authors. Any disagreement between them was discussed and agreement was reached in the end.

### Quality assessment

Newcastle-Ottawa Scale (NOS) was used to assess the methodological quality of all the included studies^13^. Three categories, including selection, comparability and exposure (case-control study) or outcomes (cohort study), were the judging criteria of the NOS. Next, it was divided into 9 items, which included the following groups; S1: Definition of cases; S2: Representativeness of the cases; S3: Selection of controls; S4: Adequate control definition; C1: Comparability of cases; C2: Study controls for the basis of the analysis; E1: Ascertainment of the exposure; E2: Ascertainment of the same method used for cases and controls; E3: Non-response rate. Each item could achieve one score if the study met the criteria. The score of a study below 6 means low quality, 6 and 7 represent moderate quality, while 8 and 9 signify good quality.

The two authors assessed the quality of the included studies, separately. Any disagreement between them was discussed and agreement was reached in the end.

### Data synthesis and analysis

All analyses were conducted by the software RevMan (Review Manager, version 5.3). Mean difference (MD) was used to report results, with a 95% CI for continuous variables. Forest plot, chi-squared homogeneity test and Higgins index (I^2^) were applied to evaluate the heterogeneity of articles. Heterogeneity was regarded as: none (I^2^<25%), low (25%≤ I^2^ <50%), moderate (25%≤ I^2^ <75%), or high (I^2^>75%). In the case of heterogeneity (chi-squared p<0.05 or I^2^>50%), the random effects model was preferred^14^. Impact of study population on the overall findings was detected by subgroup analysis. One-study removed method was used to determine the sensitivity of the meta-analysis.

## RESULTS

### Study characteristics

**Figure 1:**
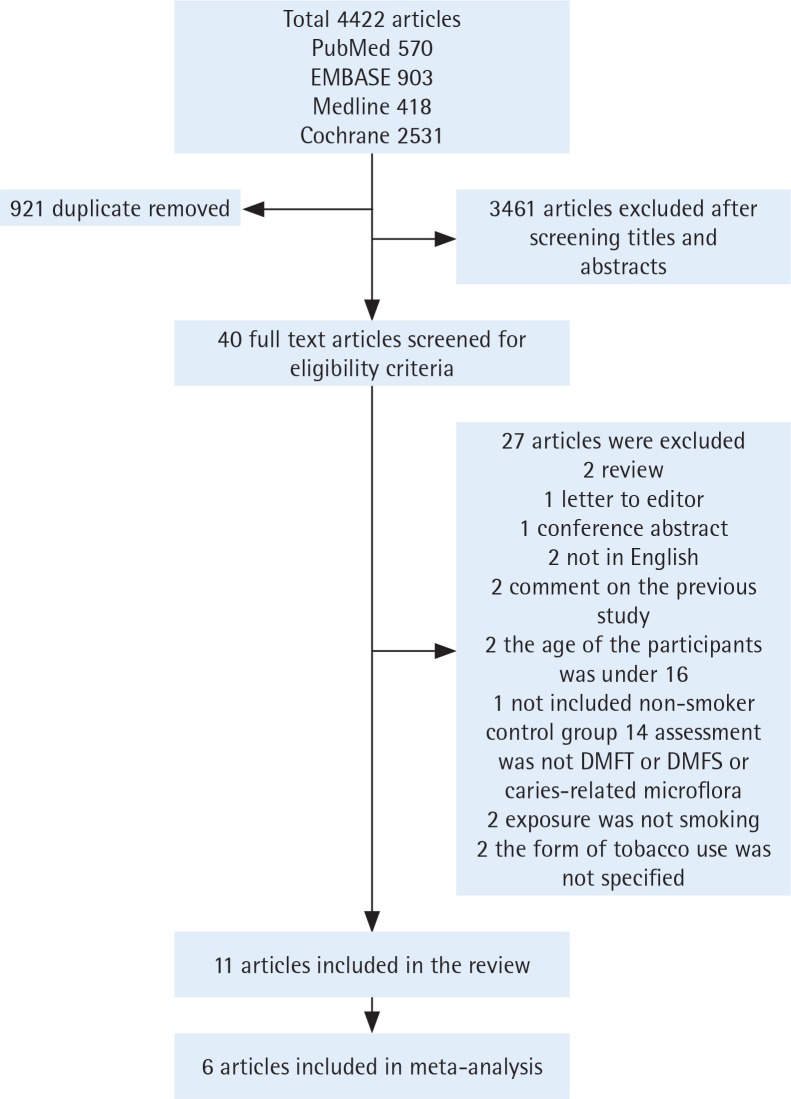
Flow chart of studies selection process

Electronic searches yielded 4422 hits, of which 921 duplicate articles were removed. Subsequently, 3461 irrelevant articles were excluded after screening the titles and abstracts. Ultimately, forty full-text articles were accessed and eleven articles were included in the systematic review. Due to different outcome formats, five studies of DMFT were included in a meta-analysis and two of DMFS in another meta-analysis. It is worth noting that one study was included twice. Finally, there were six studies included in the final meta-analysis (Figure 1). The inter-reviewer reliability was calculated by Cohen’s kappa (Kappa score = 0.88). A systematic review of the eleven appraised studies is reported in Table 1. Among them, ten^11,15-23^ used cross-sectional format and one^24^ used longitudinal format.

**Table 1:** Characteristics of the included studies

*Authors, year*	*Place*	*Study type*	*Total number (F/M)*	*Mean age (range in years)*	*Exposure assessment*	*Assessment of caries*	*Results*	*Association*
Axelsson et al.^11^ (1998)	Varmland Sweden	CS	1093 (557/536)	NR	Self-report	DMFT, DMFS	Smokers & non-smokers (35, 50, 65 and 75 years old) DS: p=0.183, 0.516, 0.122, 0.746, respectively; MS: p=0.145, 0.013, 0.007, 0.005, respectively; FS: p=0.021, 0.732, 0.012, 0.075, respectively	yes
AguilarZinser et al.^15^ (2008)	Mexico	CS	824 (All male)	35.5±10 (NR)	Self-report	DMFT, DT, MT, FT	Smokers & former smokers & non-smokers DMFT: (8.80±6.56 vs 9.86±6.05 vs 8.55±5.72)	yes
Vellappally et al.^21^ (2008)	India	CS	805 (295/580)	NR (30–69)	Self-report	DT, MT, FT	Regular smokers & occasional smokers & ex-smokers & non-tobacco users DT: (6.44±3.95 vs 3.6±2.67 vs 5.5±3.78 vs 5.1±4.25); MT: (1.9±2.14 vs1.57±2.01 vs 1.62±1.84 vs 1.53±1.65); FT: (3.29±3.2 vs 1.97±2.20 vs 3.23±3.09 vs 2.33±2.86)	yes
Campus et al.^22^ (2011)	Italia	CS	762 (41/721)	24.7±3.8 (NR)	Self-report	DMFS, DS, FS, MS	Heavy smokers & light smokers & non-smokers DMFS: (11.5±0.7 vs 11.3±0.6 vs 9.9±0.8)	yes
Rwenyonyi et al.^23^ (2011)	Rakai District Uganda	CS	321 (152/169)	38.8±15.5 (18–62)	Self-report	DMFT	Spearman’s rank correlation coefficients between tobacco smoking and DMFT scores = 0.28 (a statistically significant)	yes
Badel et al.^16^ (2014)	Koprivnica	CS	505 (All male)	19 (NR)	Self-report	DMFT, F-ST	Smokers & non-smokers DT: (3.58±3.45 vs 2.56±2.79, p<0.001); FT: (2.51±3.33 vs 3.41±3.79, p=0.005); F-ST: (23.11±4.41 vs 24.19±3.62, p=0.004)	yes
Bernabe et al.^24^ (2014)	Finland	L(p)	955 (520/435)	48.4±11.9 (30–89)	Self-report	DMFT, FT, MT, DT	DT increment: IRR (95% CI) = 1.70 (1.07–2.70); While daily smoking was not associated with FT, MT and DMFT increment.	yes
Tanner et al.^17^ (2014)	Finland	CS	8537 (All male)	19.6 (NR)	Self-report	DMTF, DT	Smokers & non-smokers DMFT: (5.43±4.85 vs 3.55±3.78) DT: (2.23±3.29 vs 1.07±2.05)	yes
Tanner et al.^18^ (2015)	Finland	CS	8539 (All male)	19.6 (NR)	Self-report	DMFT, DT	Smokers & non-smokers DMFT: (6.35±4.86 vs 3.75±4.05) DT: (2.37±3.26 vs 1.15±2.18)	yes
NakoniecznaRudnicka et al.^20^ (2017)	Lublin	CS	116 (76/40)	30.7±10.3 (NR)	Self-report and cotinine test	CRT bacteria test	Smokers & non-smokers SM bacteria: χ2=1.58 (-) p>0.05 LB bacteria: χ2=0.45 (-) p>0.05	no
Sharma et al.^19^ (2018)	India	CS	300 (All Male)	NR (20–40)	Self-report	DMFT, DMFS	Smokers & non-smokers DMFT: (2.50±1.514 vs 1.75±1.417) DMFS: (5.67±4.195 vs 3.18±3.056)	yes

CS: cross-sectional, L(p): longitudinal (prospective), F: female, M: male, NR: not reported, DMFT: decayed, missing and filled teeth, DMFS: decayed, missing and filled surface, DS: decayed surface, MS: missing surface, FS: filled surface, DT: decayed teeth, MT: missing teeth, FT: filled teeth, FS-T: filled and sound teeth, IRR: incidence rate ratios, CI: confidence interval.

All the participants were adults. Six studies sampled both males and females, while five only included males. One study^20^ assessed smoking using self-report and cotinine tests, the remaining studies used self-report only. Seven studies^15-21,23^ analyzed DMFT and one^22^ analyzed DMFS. Two studies^11,19^ accessed both. One study^20^ reported the levels of *Streptococcus mutans* (SM) and *Lactobacillus* (LB).

### Meta-analysis

Only studies that used mean value and standard deviation (SD) as data representations of DMFT/DMFS were included in the meta-analysis.

**Figure 2:**
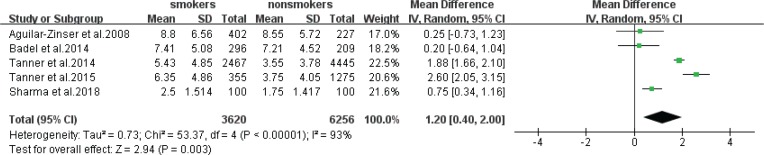
Pooled effect of smoking on caries in the form of DMFT

Five statistical results using DMFT as outcome format were included in a meta-analysis (Figure 2). The estimate obtained via the random effects model was statistically significant (z-test p=0.003), with an MD of 1.20 (95% CI: 0.40–2.00), which meant that the prevalence of caries in smokers was higher than that of non-smokers. The heterogeneity was high (I^2^=93%, chi-squared p<0.00001).

**Figure 3:**
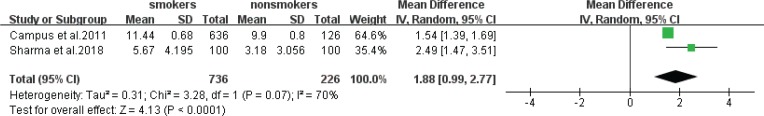
Pooled effect of smoking on caries in the form of DMFS

Data of DMFS extracted from two studies were also analyzed (Figure 3). The estimate obtained via the random effects model was also statistically significant (z-test p<0.0001) with an MD of 1.88 (95% CI: 0.99–2.77) indicating that the prevalence of cavities in smokers was significantly higher than that of non-smokers. The heterogeneity was also high (I^2^=70%, chi-squared p=0.07).

### Subgroup and sensitivity analyses

DMFT

In subgroup analysis of study samples, only the randomly selected population showed significant differences, while the specific ones did not. Sensitivity analysis of DMFT through the one-study removed method did not find any study that affected the heterogeneity (Table 2). There was no significant decrease in the heterogeneity of subgroup analysis and sensitivity analysis, so it was impossible to find the source of heterogeneity. More research is needed to confirm the conclusion.

**Table 2:** Results of subgroup and sensitivity analyses

*DMFT*	*Number of studies*	*Heterogeneity*		*Model*	*Meta-analysis*		
		*I^2^ (%)*	*p*		*MD*	*95% CI*	*p*
**Subgroup analyses (study population)**							
Random sample	3	94	<0.00001	RE	1.73	0.83–2.63	0.0002
Army recruits	1	NA	NA	NA	0.20	-0.64–1.64	0.64
Drivers	1	NA	NA	NA	0.25	-0.73–1.23	0.62
**Sensitivity analyses**							
Study by Aguilar-Zinser et al. omitted	4	93	<0.00001	RE	1.40	0.54–2.25	0.01
Study by Badel et al. omitted	4	93	<0.00001	RE	1.43	0.59–2.27	0.0009
Study by Tanner et al. in 2014 omitted	4	92	<0.00001	RE	0.98	-0.16–2.13	0.09
Study by Tanner et al. in 2015 omitted	4	92	<0.00001	RE	0.84	-0.06–1.74	0.07
Study by Sharma et al. omitted	4	91	<0.00001	RE	1.32	0.39–2.25	0.005

DMFT: decayed, missing and filled teeth, NA: not available, RE: random effects, MD: mean difference, CI: confidence interval.

DMFS

Since DMFS data reported by only two articles were available, a sensitivity analysis was not conducted.

### Quality assessment

The scores of all studies ranged from 7 to 9 (Table 3). Many of them lacked the representativeness of the cases and did not take adequate actions to avoid the bias of the study analysis. Despite that, all had good quality (Kappa score = 0.80).

**Table 3:** Quality of the studies was assessed by the Newcastle-Ottawa scale

*Study*	*Selection*				*CMP*		*Exposure/Outcome*			*Total stars*

	*S1*	*S2*	*S3*	*S4*	*C1*	*C2*	*E1*	*E2*	*E3*	
Axelsson et al.^11^ (1998)	*	*	*	*	*		*	*		7
Aguilar-Zinser et al.^15^ (2008)	*		*	*	*	*	*	*	*	8
Vellappally et al.^21^ (2008)	*	*	*	*	*	*	*	*	*	9
Campus et al.^22^ (2011)	*		*	*	*	*	*	*	*	8
Rwenyonyi et al.^23^ (2011)	*		*	*	*	*	*	*	*	8
Badel et al.^16^ (2014)	*		*	*	*		*	*	*	7
Bernabe et al.^24^ (2014)	*	*	*	*	*	*	*	*		8
Tanner et al.^17^ (2014)	*		*	*	*		*	*	*	7
Tanner et al.^18^ (2015)	*	*	*	*	*	*	*	*	*	9
Nakonieczna-Rudnicka et al.^20^ (2017)	*	*	*	*	*	*	*	*	*	9
Sharma et al.^19^ (2018)	*		*	*	*		*	*	*	7

CMP: Comparability, C1: Comparability of cases, C2: Study controls for the basis of the analysis, S1: Definition of cases, S2: Representativeness of the cases, S3: Selection of controls, S4: Adequate control definition, E1: Ascertainment of the exposure, E2: Ascertainment of the same method used for cases and controls, E3: Non-response rate.

## DISCUSSION

Findings from this systematic review and meta-analysis indicate the existence of a relation between tobacco smoking and dental caries. For most of the studies reviewed, the results were consistent with a positive association. Except for one longitudinal study by Bernabe et al.^24^, most were cross-sectional and thus do not allow inferences to be made on causal relations; affirmation of the etiology of tobacco smoking was not possible. Therefore, there was insufficient evidence to confirm the hypothesis that tobacco, as a risk factor, is involved in the dental caries process. More longitudinal studies are needed to come to any conclusion.

One study conducted by Nakonieczna-Rudnicka et al.^20^ assessed the amount of SM and LB in the saliva of non-smokers and smokers. They concluded that there was no essential correlation between the number of SM and LB and the number of cigarettes smoked per day, the duration of smoking and the smoking status. However, some experiments have studied the effect of nicotine on SM. A study conducted by Chanea et al.^25^ found that SM adherence was significantly enhanced in the presence of nicotine. SM makes use of sucrose for metabolism, and its byproducts are mainly responsible for adherence and caries generation. In addition, Ashkanane et al.^26^ and Mohammed et al.^27^ have examined the effect of nicotine and cigarette extracts on oral bacteria. In a recent review^28^ on the effects of nicotine on oral microorganisms and human tissues there is indirect evidence of a link between smoking and caries. Taken together, more research is needed to confirm the association between smoking and cariogenic bacteria growth.

### Limitations

Several limitations may influence the results of the systematic review and meta-analysis. First, most of the articles included were cross-sectional studies that could only judge whether there was a connection between smoking and dental caries, but could not determine the causal relationship between them.

Second, is the subjective bias associated with questionnaire surveys. Participants were aware of the purpose of the investigation in all the studies. However, as it is generally accepted that smoking is harmful to health, the participants may have given socially acceptable responses, especially in front of the medical staff. Only one study included both saliva cotinine test and the questionnaire survey. Cotinine is a nicotine breakdown product used to determine whether people smoke. Therefore, the actual number of smokers and the severity of smoking may be higher than the survey results, leading to biased results on the relationship between tobacco smoking and dental caries.

Third, is the specific population and gender covered in trials. The prevalence of dental caries is impacted upon by different lifestyle habits, regional development level, special occupation, education, expenditure for dental care, age, gender etc. Most of the research only provided classified statistics, without making adjustment when they analyzed the association between tobacco smoking and caries. In addition, the study populations included professional Mexican truck drivers^15^, Croatian army recruits^16^, and people in the Italian military academy^22^, all of whom were unrepresentative. Moreover, five statistical results using DMFT as outcome format were included in a meta-analysis, but the study group was all male. In summary, the overall representativeness of studies is not good so there are some deviations in the conclusions.

Forth, is the completeness of the studies. As the search strategy part stated, the literature search is limited and there may be some omissions, as well as not taking into account Masters and PhD theses etc. Beyond that, there were only five studies of DMFT and two of DMFS included in our analysis, so we have not conducted funnel plots because it is advised to analyse at least ten studies^29^.

## CONCLUSIONS

In light of the above, there is a correlation between tobacco smoking and an increased risk of dental caries. However, the overall representativeness of the selected studies is not good. More prospective and extensive studies on this topic are needed in the future to get validation, and they will require to adopt both a questionnaire survey and detection of cotinine in saliva, define the specific severity of smoking, distinguish between smoking types, adjust the extraneous variables, select representative groups etc. Nevertheless, it is imperative for people to cease tobacco smoking.

## References

[1] (2014). The Health Consequences of Smoking- 50 Years of Progress: A Report of the Surgeon General.

[2] World Health Organization (2017). WHO report on the global tobacco epidemic, 2017: monitoring tobacco use and prevention policies.

[3] Mitali R, Aarti G, Pramod Y, Kunal J, Sahil H (2016). Diagnostic Methods for Detection of Cotinine Level in Tobacco Users: A Review. Journal of Clinical and Diagnostic Research.

[4] Mainali P, Pant S, Rodriguez A P (2015). Tobacco and Cardiovascular Health. Cardiovascular Toxicology.

[5] Petersen PE (2003). Tobacco and oral health – the role of the World Health Organization. Oral Health Prev Dent.

[6] Selwitz RH, Ismail AI, Pitts N B (2007). Dental caries. Lancet.

[7] Islam B, Khan SN, Khan AU (2007). Dental caries: from infection to prevention. Medical Science Monit.

[8] Kaewkamnerdpong I, Krisdapong S (2018). The Associations of School Oral Health-Related Environments with Oral Health Behaviours and Dental Caries in Children. Caries Research.

[9] Gregoire C (2010). How to manage odontogenic infections. Todays Fda Official Monthly Journal of the Florida Dental Association.

[10] Gupta P, Gupta N, Singh HP (2014). Prevalence of Dental Caries in relation to Body Mass Index, Daily Sugar Intake, and Oral Hygiene Status in 12-Year-Old School Children in Mathura City: A Pilot Study. Int J Pediatr.

[11] Axelsson P, Paulander J, Lindhe J (1998). Relationship between smoking and dental status in 35-, 50-, 65-, and 75-year-old individuals. Journal Of Clinical Periodontology.

[12] Moher D, Liberati A, Tetzlaff J (2009). Preferred Reporting Items for Systematic Reviews and Meta-Analyses: The PRISMA Statement. PLoS Medicine.

[13] Stang A (2010). Critical evaluation of the Newcastle-Ottawa scale for the assessment of the quality of nonrandomized studies in meta-analyses. European Journal of Epidemiology.

[14] Leite FRM, Nascimento GG, Scheutz F (2018). Effect of Smoking on Periodontitis: A Systematic Review and Meta-regression. American Journal of Preventive Medicine.

[15] Aguilar-Zinser V, Irigoyen ME, Rivera G (2008). Cigarette Smoking and Dental Caries among Professional Truck Drivers in Mexico. Caries Research.

[16] Badel T, Pavicin IS, Carek AJ (2014). Dental caries experience and tobacco use in 19-year-old Croatian army recruits. Coll Antropol.

[17] Tanner T, Antti Kämppi, Jari Päkkilä (2014). Association of smoking and snuffing with dental caries occurrence in a young male population in Finland: A cross-sectional study. Acta Odontologica Scandinavica.

[18] Tanner T, Päkkilä J, Karjalainen K (2015). Smoking, alcohol use, socioeconomic background and oral health among young Finnish adults. Community Dentistry and Oral Epidemiology.

[19] Sharma S, Mishra SK, Mittal N (2018). Influence of tobacco dependence on caries development in young male adults: A cross-sectional study. Journal of Conservative Dentistry.

[20] Nakonieczna-Rudnicka M, Bachanek T (2017). Number of Streptococcus mutans and Lactobacillus in saliva versus the status of cigarette smoking, considering duration of smoking and number of cigarettes smoked daily. Ann Agric Environ Med.

[21] Vellappally S, Jacob V, Smejkalová J, Shriharsha P, Kumar V, Fiala Z (2008). Tobacco habits and oral health status in selected Indian population. Cent Eur J Public Health.

[22] Campus G, Cagetti MG, Senna A (2011). Does smoking increase risk for caries? a cross-sectional study in an Italian military academy. Caries Research.

[23] Rwenyonyi CM, Muwazi LM, Buwembo W (2011). Assessment of factors associated with dental caries in rural communities in Rakai District, Uganda. Clinical Oral Investigations.

[24] Bernabé Ε, Delgado-Angulo EK, Vehkalahti MM (2014). Daily smoking and 4-year caries increment in Finnish adult. Community Dentistry and Oral Epidemiology.

[25] Chanea Κ, Palmire A (2014). Smoking and Candy on Oral Bacteria, Streptococcus mutans, Adherence. California State Polytechnic University Pomona.

[26] Ashkanane A, Gomez GF, Levon J, Windsor LJ, Eckert GJ, Gregory RL (2017). Nicotine Upregulates Coaggregation of Candida albicans and Streptococcus mutans. Journal of Prosthodontics Official Journal of the American College of Prosthodontists.

[27] Mohammed AY, Gomez GF, Eckert GJ (2018). The Impact of Nicotine and Cigarette Smoke Condensate on Metabolic Activity and Biofilm Formation of\r, Candida albicans\r, on Acrylic Denture Material. Journal of Prosthodontics.

[28] Wagenknecht DR, Balhaddad AB, Gregory RL (2018). Effects of Nicotine on Oral Microorganisms, Human Tissues, and the Interactions between Them. Current Oral Health Reports.

[29] Higgins JPT, Green S (2011). Cochrane Handbook for Systematic Reviews of Interventions Version 5.1.0.

